# Integrated bioinformatics and clinical data identify three novel biomarkers for osteoarthritis diagnosis and synovial immune

**DOI:** 10.1038/s41598-025-95837-7

**Published:** 2025-03-31

**Authors:** Zheng Zhu, Bizhi Tu, Cheng Peng, Xun Xu, Peizhi Lu, Rende Ning

**Affiliations:** Department of Orthopedics, Hefei First People’s Hospital, Anhui Medical University, 390 Huaihe Road, Hefei, 230061 Anhui China

**Keywords:** Osteoarthritis, Biomarker, Diagnostic model, Immune infiltration, Bioinformatics, Immunological disorders, Metabolic disorders, Diagnostic markers, Predictive markers, Prognostic markers, Immunology, Biomarkers, Diseases, Medical research, Risk factors

## Abstract

Osteoarthritis (OA) is a degenerative joint disease that can be aggravated by synovitis and synovial immune disorders (SID). However, the role of synovial SID-related genes in OA synovium remains poorly understood. OA synovial and peripheral blood datasets were obtained from the GEO database (https://www.ncbi.nlm.nih.gov/). Immune-related genes (https://reactome.org/) showing differential expression in peripheral blood were identified as immune disorder genes. Subsequently, differentially expressed immune disorder genes in OA synovium were further identified as SID genes. The Venn diagram, random forest, SVM-RFE algorithm, and multivariate analysis were employed to determine SID-related hub genes in OA synovium. Using the identified hub genes, we constructed and validated a diagnostic model for predicting OA occurrence. The correlation between hub gene expression and immune-related modules was explored using CIBERSORT and MCP-counter analyses. We identified three SID-related hub genes (*ACAT1*, *SPHK1*, and *ACACB*) in OA synovium. The diagnostic model incorporating these hub genes demonstrated reliable predictive accuracy (AUC = 0.939). Through qPCR analysis, we quantitated the expression levels of the hub genes and confirmed that three hub genes could serve as novel biomarkers for OA patients (AUC = 0.960). Furthermore, we observed a significant correlation between the expression of these hub genes and immune cell infiltration, as well as inflammatory cytokine levels in OA synovium. Our findings suggest that three SID-related hub genes have the potential to serve as diagnostic biomarkers for OA patients. These genes are associated with immune disorder and contribute to immune alterations within the OA synovium.

## Introduction

Osteoarthritis (OA) is a degenerative joint disease characterized by clinical manifestations, including joint pain, functional impairment, and structural alterations^1^. Various factors, such as age, dyslipidemia, synovitis, and genetic factors^2–4^, contribute to the progression of OA. With over 250 million estimated cases worldwide, OA poses a significant socioeconomic burden each year^5–7^. Currently, available treatment options for early-stage OA are limited to nonsteroidal anti-inflammatory drugs and physical therapy, aimed at slowing down disease progression^8^. However, as OA advances, joint replacement becomes inevitable^9^. Unfortunately, early symptoms of OA often go unnoticed, causing some patients to miss the optimal window for early intervention^10^. While previous studies have reported potential early diagnostic biomarkers for OA^11^, their testing may be challenging as they require samples from joint components (such as cartilage, synovium, meniscus, or subchondral bone) that are not suitable for routine screening purposes^12^. Therefore, the development of a reliable blood marker for early diagnosis of OA would hold great clinical utility.

Mounting evidence suggests that OA is not solely a mechanical disease characterized by meniscus degeneration, subchondral sclerosis, and cartilage wear, but also involves immune system dysregulation and inflammation^2,4,13^. In OA, pathological changes in the synovium may precede those in the cartilage, with the severity of synovial lesions worsening as the disease progresses^14–16^. Synovial immune dysfunction has been identified as a crucial factor in OA pathogenesis^17^.Various immune cells within PBMCs (such as T cells, B cells, and macrophages) and inflammatory factors (including IL-2, IL-10, and IFN-γ) are recruited to the synovial tissue, triggering an inflammatory cascade that exacerbates synovitis and contributes to OA progression^18,19^. Thus, immune dysregulation within the synovial region is marked by substantial infiltration of immune cells and inflammatory mediators^20^. The affected synovial tissue can directly or indirectly drive changes in the polarization of blood-derived macrophages^21^, as well as alter the activation and differentiation potential of T cells^22^, B cells^23^, and other immune cells. While these observations highlight a close interplay between synovial immune dysregulation and peripheral blood cells, the expression levels of synovial immune-related genes in PBMCs and their clinical importance remain undefined.

In this study, we investigated the SID-related genes that differentially expressed both in blood and synovium samples from OA patients. Furthermore, we explored whether SID related hub genes could serve as diagnostic markers for predicting the occurrence of OA. Our findings demonstrate that the three SID related hub genes can serve as reliable blood markers for diagnosing OA. Moreover, the expression of these hub genes may be implicated in the dysregulation of synovial immune responses in OA patients.

## Method

### Processing of raw data

**Fig. 1:**
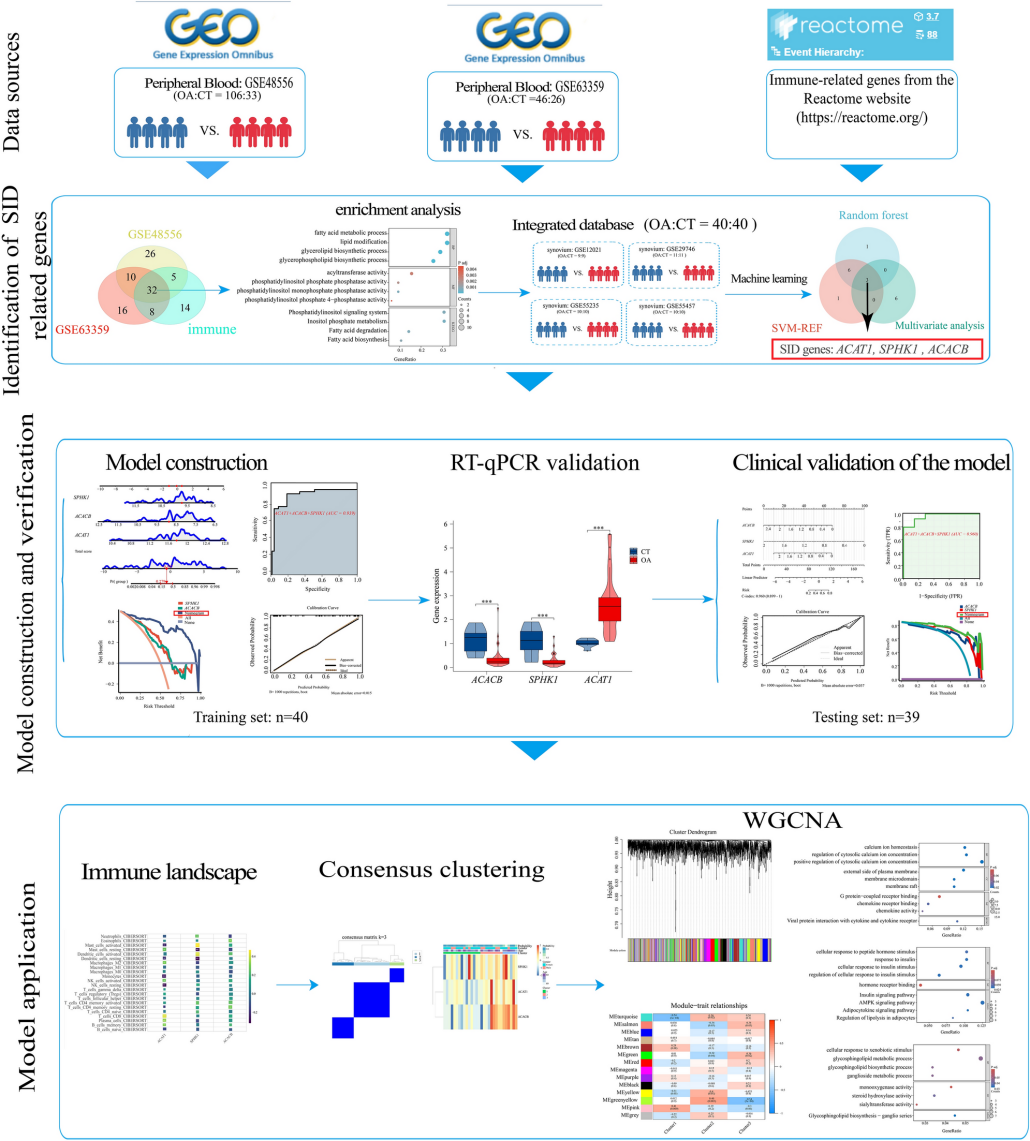
The work flow chart.

The workflow chart of this study is shown in (Fig. 1). Four synovium (GSE12021, GSE29746, GSE55235, GSE55457) and PBMC (GSE48556, GSE63359) datasets of patients with OA were downloaded from the GEO database (https://www.ncbi.nlm.nih.gov/geo/). The detailed information for these six GEO datasets is present in (Table 1). Gene annotation was performed according to the annotation files provided by the corresponding platforms. Probes without matching gene symbols were removed. For multiple probes matching the same gene symbol, the mean value was used. The SVA package was used to remove the batch effect and then obtained an integrated synovium-related database (training set, containing 40 OA and 40 healthy synovial samples). We obtained the immune-related genes from the Reactome website (https://reactome.org/) and these genes that are differential expressed in peripheral blood in OA patients were identified as SID genes.

**Table 1:** Summarize GEO data information included in the study.

Database ID	Sample ID	Organism	Age (year)	Sex	Group
GSE48556	GSM1182292	Homo sapiens	50	Female	CT
GSE48556	GSM1182293	Homo sapiens	68	Female	CT
GSE48556	GSM1182294	Homo sapiens	76	Female	CT
GSE48556	GSM1182295	Homo sapiens	66	Female	CT
GSE48556	GSM1182296	Homo sapiens	60	Female	CT
GSE48556	GSM1182297	Homo sapiens	57	Male	CT
GSE48556	GSM1182298	Homo sapiens	63	Female	CT
GSE48556	GSM1182299	Homo sapiens	47	Female	CT
GSE48556	GSM1182300	Homo sapiens	54	Female	CT
GSE48556	GSM1182301	Homo sapiens	60	Female	CT
GSE48556	GSM1182302	Homo sapiens	59	Male	CT
GSE48556	GSM1182303	Homo sapiens	60	Female	CT
GSE48556	GSM1182304	Homo sapiens	62	Female	CT
GSE48556	GSM1182305	Homo sapiens	59	Female	CT
GSE48556	GSM1182306	Homo sapiens	46	Female	CT
GSE48556	GSM1182307	Homo sapiens	57	Female	CT
GSE48556	GSM1182308	Homo sapiens	68	Male	CT
GSE48556	GSM1182309	Homo sapiens	62	Female	CT
GSE48556	GSM1182310	Homo sapiens	62	Male	CT
GSE48556	GSM1182311	Homo sapiens	62	Male	CT
GSE48556	GSM1182312	Homo sapiens	47	Female	CT
GSE48556	GSM1182313	Homo sapiens	51	Male	CT
GSE48556	GSM1182314	Homo sapiens	54	Female	CT
GSE48556	GSM1182315	Homo sapiens	59	Male	CT
GSE48556	GSM1182316	Homo sapiens	63	Female	CT
GSE48556	GSM1182317	Homo sapiens	65	Male	CT
GSE48556	GSM1182318	Homo sapiens	44	Female	CT
GSE48556	GSM1182319	Homo sapiens	48	Female	CT
GSE48556	GSM1182320	Homo sapiens	50	Female	CT
GSE48556	GSM1182321	Homo sapiens	64	Female	CT
GSE48556	GSM1182322	Homo sapiens	60	Female	CT
GSE48556	GSM1182323	Homo sapiens	51	Male	CT
GSE48556	GSM1182324	Homo sapiens	67	Female	CT
GSE48556	GSM1182186	Homo sapiens	48	Female	OA
GSE48556	GSM1182187	Homo sapiens	60	Female	OA
GSE48556	GSM1182188	Homo sapiens	63	Female	OA
GSE48556	GSM1182189	Homo sapiens	60	Female	OA
GSE48556	GSM1182190	Homo sapiens	70	Female	OA
GSE48556	GSM1182191	Homo sapiens	50	Female	OA
GSE48556	GSM1182192	Homo sapiens	47	Female	OA
GSE48556	GSM1182193	Homo sapiens	63	Female	OA
GSE48556	GSM1182194	Homo sapiens	75	Female	OA
GSE48556	GSM1182195	Homo sapiens	78	Female	OA
GSE48556	GSM1182196	Homo sapiens	53	Female	OA
GSE48556	GSM1182197	Homo sapiens	57	Female	OA
GSE48556	GSM1182198	Homo sapiens	73	Female	OA
GSE48556	GSM1182199	Homo sapiens	77	Female	OA
GSE48556	GSM1182200	Homo sapiens	65	Female	OA
GSE48556	GSM1182201	Homo sapiens	64	Female	OA
GSE48556	GSM1182202	Homo sapiens	57	Female	OA
GSE48556	GSM1182203	Homo sapiens	55	Female	OA
GSE48556	GSM1182204	Homo sapiens	54	Female	OA
GSE48556	GSM1182205	Homo sapiens	64	Female	OA
GSE48556	GSM1182206	Homo sapiens	65	Female	OA
GSE48556	GSM1182207	Homo sapiens	58	Female	OA
GSE48556	GSM1182208	Homo sapiens	59	Female	OA
GSE48556	GSM1182209	Homo sapiens	58	Female	OA
GSE48556	GSM1182210	Homo sapiens	56	Female	OA
GSE48556	GSM1182211	Homo sapiens	59	Female	OA
GSE48556	GSM1182212	Homo sapiens	60	Female	OA
GSE48556	GSM1182213	Homo sapiens	57	Female	OA
GSE48556	GSM1182214	Homo sapiens	62	Female	OA
GSE48556	GSM1182215	Homo sapiens	54	Female	OA
GSE48556	GSM1182216	Homo sapiens	79	Female	OA
GSE48556	GSM1182217	Homo sapiens	62	Female	OA
GSE48556	GSM1182218	Homo sapiens	56	Female	OA
GSE48556	GSM1182219	Homo sapiens	56	Female	OA
GSE48556	GSM1182220	Homo sapiens	65	Female	OA
GSE48556	GSM1182221	Homo sapiens	59	Female	OA
GSE48556	GSM1182222	Homo sapiens	59	Female	OA
GSE48556	GSM1182223	Homo sapiens	64	Female	OA
GSE48556	GSM1182224	Homo sapiens	71	Female	OA
GSE48556	GSM1182225	Homo sapiens	60	Female	OA
GSE48556	GSM1182226	Homo sapiens	57	Female	OA
GSE48556	GSM1182227	Homo sapiens	56	Female	OA
GSE48556	GSM1182228	Homo sapiens	50	Female	OA
GSE48556	GSM1182229	Homo sapiens	51	Female	OA
GSE48556	GSM1182230	Homo sapiens	53	Female	OA
GSE48556	GSM1182231	Homo sapiens	71	Female	OA
GSE48556	GSM1182232	Homo sapiens	57	Female	OA
GSE48556	GSM1182233	Homo sapiens	71	Female	OA
GSE48556	GSM1182234	Homo sapiens	59	Female	OA
GSE48556	GSM1182235	Homo sapiens	65	Female	OA
GSE48556	GSM1182236	Homo sapiens	79	Female	OA
GSE48556	GSM1182237	Homo sapiens	66	Female	OA
GSE48556	GSM1182238	Homo sapiens	63	Female	OA
GSE48556	GSM1182239	Homo sapiens	67	Female	OA
GSE48556	GSM1182240	Homo sapiens	46	Female	OA
GSE48556	GSM1182241	Homo sapiens	43	Female	OA
GSE48556	GSM1182242	Homo sapiens	57	Female	OA
GSE48556	GSM1182243	Homo sapiens	60	Female	OA
GSE48556	GSM1182244	Homo sapiens	70	Female	OA
GSE48556	GSM1182245	Homo sapiens	61	Female	OA
GSE48556	GSM1182246	Homo sapiens	68	Female	OA
GSE48556	GSM1182247	Homo sapiens	54	Female	OA
GSE48556	GSM1182248	Homo sapiens	57	Female	OA
GSE48556	GSM1182249	Homo sapiens	59	Female	OA
GSE48556	GSM1182250	Homo sapiens	68	Female	OA
GSE48556	GSM1182251	Homo sapiens	67	Female	OA
GSE48556	GSM1182252	Homo sapiens	61	Female	OA
GSE48556	GSM1182253	Homo sapiens	62	Female	OA
GSE48556	GSM1182254	Homo sapiens	53	Female	OA
GSE48556	GSM1182255	Homo sapiens	60	Female	OA
GSE48556	GSM1182256	Homo sapiens	55	Female	OA
GSE48556	GSM1182257	Homo sapiens	46	Female	OA
GSE48556	GSM1182258	Homo sapiens	58	Female	OA
GSE48556	GSM1182259	Homo sapiens	60	Female	OA
GSE48556	GSM1182260	Homo sapiens	58	Female	OA
GSE48556	GSM1182261	Homo sapiens	74	Female	OA
GSE48556	GSM1182262	Homo sapiens	74	Female	OA
GSE48556	GSM1182263	Homo sapiens	59	Female	OA
GSE48556	GSM1182264	Homo sapiens	62	Female	OA
GSE48556	GSM1182265	Homo sapiens	68	Female	OA
GSE48556	GSM1182266	Homo sapiens	57	Female	OA
GSE48556	GSM1182267	Homo sapiens	64	Female	OA
GSE48556	GSM1182268	Homo sapiens	58	Female	OA
GSE48556	GSM1182269	Homo sapiens	53	Female	OA
GSE48556	GSM1182270	Homo sapiens	55	Female	OA
GSE48556	GSM1182271	Homo sapiens	59	Female	OA
GSE48556	GSM1182272	Homo sapiens	61	Female	OA
GSE48556	GSM1182273	Homo sapiens	59	Female	OA
GSE48556	GSM1182274	Homo sapiens	48	Male	OA
GSE48556	GSM1182275	Homo sapiens	67	Female	OA
GSE48556	GSM1182276	Homo sapiens	69	Female	OA
GSE48556	GSM1182277	Homo sapiens	74	Female	OA
GSE48556	GSM1182278	Homo sapiens	67	Female	OA
GSE48556	GSM1182279	Homo sapiens	75	Female	OA
GSE48556	GSM1182280	Homo sapiens	77	Female	OA
GSE48556	GSM1182281	Homo sapiens	60	Female	OA
GSE48556	GSM1182282	Homo sapiens	62	Female	OA
GSE48556	GSM1182283	Homo sapiens	58	Female	OA
GSE48556	GSM1182284	Homo sapiens	61	Female	OA
GSE48556	GSM1182285	Homo sapiens	53	Female	OA
GSE48556	GSM1182286	Homo sapiens	55	Female	OA
GSE48556	GSM1182287	Homo sapiens	54	Female	OA
GSE48556	GSM1182288	Homo sapiens	51	Female	OA
GSE48556	GSM1182289	Homo sapiens	60	Female	OA
GSE48556	GSM1182290	Homo sapiens	66	Female	OA
GSE48556	GSM1182291	Homo sapiens	59	Female	OA
GSE63359	GSM1546587	Homo sapiens	46	Female	CT
GSE63359	GSM1546588	Homo sapiens	54	Female	CT
GSE63359	GSM1546589	Homo sapiens	54	Female	CT
GSE63359	GSM1546590	Homo sapiens	56	Male	CT
GSE63359	GSM1546591	Homo sapiens	54	Female	CT
GSE63359	GSM1546592	Homo sapiens	52	Male	CT
GSE63359	GSM1546593	Homo sapiens	62	Female	CT
GSE63359	GSM1546594	Homo sapiens	49	Female	CT
GSE63359	GSM1546595	Homo sapiens	55	Female	CT
GSE63359	GSM1546596	Homo sapiens	49	Female	CT
GSE63359	GSM1546597	Homo sapiens	56	Female	CT
GSE63359	GSM1546598	Homo sapiens	57	Male	CT
GSE63359	GSM1546599	Homo sapiens	49	Female	CT
GSE63359	GSM1546600	Homo sapiens	58	Female	CT
GSE63359	GSM1546601	Homo sapiens	66	Female	CT
GSE63359	GSM1546602	Homo sapiens	31	Female	CT
GSE63359	GSM1546603	Homo sapiens	58	Female	CT
GSE63359	GSM1546604	Homo sapiens	48	Male	CT
GSE63359	GSM1546605	Homo sapiens	65	Male	CT
GSE63359	GSM1546606	Homo sapiens	50	Female	CT
GSE63359	GSM1546607	Homo sapiens	52	Male	CT
GSE63359	GSM1546608	Homo sapiens	87	Female	CT
GSE63359	GSM1546609	Homo sapiens	50	Male	CT
GSE63359	GSM1546610	Homo sapiens	49	Female	CT
GSE63359	GSM1546611	Homo sapiens	53	Female	CT
GSE63359	GSM1546612	Homo sapiens	59	Female	CT
GSE63359	GSM1546613	Homo sapiens	69	Female	OA
GSE63359	GSM1546614	Homo sapiens	74	Male	OA
GSE63359	GSM1546615	Homo sapiens	53	Female	OA
GSE63359	GSM1546616	Homo sapiens	64	Female	OA
GSE63359	GSM1546617	Homo sapiens	74	Female	OA
GSE63359	GSM1546618	Homo sapiens	52	Female	OA
GSE63359	GSM1546619	Homo sapiens	70	Female	OA
GSE63359	GSM1546620	Homo sapiens	80	Male	OA
GSE63359	GSM1546621	Homo sapiens	83	Female	OA
GSE63359	GSM1546622	Homo sapiens	77	Male	OA
GSE63359	GSM1546623	Homo sapiens	49	Female	OA
GSE63359	GSM1546624	Homo sapiens	61	Female	OA
GSE63359	GSM1546625	Homo sapiens	51	Female	OA
GSE63359	GSM1546626	Homo sapiens	75	Female	OA
GSE63359	GSM1546627	Homo sapiens	72	Male	OA
GSE63359	GSM1546628	Homo sapiens	84	Female	OA
GSE63359	GSM1546629	Homo sapiens	72	Female	OA
GSE63359	GSM1546630	Homo sapiens	66	Female	OA
GSE63359	GSM1546631	Homo sapiens	64	Female	OA
GSE63359	GSM1546632	Homo sapiens	56	Male	OA
GSE63359	GSM1546633	Homo sapiens	75	Female	OA
GSE63359	GSM1546634	Homo sapiens	56	Female	OA
GSE63359	GSM1546635	Homo sapiens	79	Female	OA
GSE63359	GSM1546636	Homo sapiens	72	Male	OA
GSE63359	GSM1546637	Homo sapiens	56	Male	OA
GSE63359	GSM1546638	Homo sapiens	65	Female	OA
GSE63359	GSM1546639	Homo sapiens	53	Female	OA
GSE63359	GSM1546640	Homo sapiens	47	Female	OA
GSE63359	GSM1546641	Homo sapiens	57	Male	OA
GSE63359	GSM1546642	Homo sapiens	50	Male	OA
GSE63359	GSM1546643	Homo sapiens	50	Female	OA
GSE63359	GSM1546644	Homo sapiens	78	Female	OA
GSE63359	GSM1546645	Homo sapiens	76	Female	OA
GSE63359	GSM1546646	Homo sapiens	52	Female	OA
GSE63359	GSM1546647	Homo sapiens	80	Male	OA
GSE63359	GSM1546648	Homo sapiens	56	Female	OA
GSE63359	GSM1546649	Homo sapiens	68	Female	OA
GSE63359	GSM1546650	Homo sapiens	62	Male	OA
GSE63359	GSM1546651	Homo sapiens	78	Female	OA
GSE63359	GSM1546652	Homo sapiens	73	Female	OA
GSE63359	GSM1546653	Homo sapiens	62	Female	OA
GSE63359	GSM1546654	Homo sapiens	57	Male	OA
GSE63359	GSM1546655	Homo sapiens	74	Male	OA
GSE63359	GSM1546656	Homo sapiens	74	Male	OA
GSE63359	GSM1546657	Homo sapiens	69	Female	OA
GSE63359	GSM1546658	Homo sapiens	54	Female	OA
GSE12021	GSM302859	Homo sapiens	61	Male	CT
GSE12021	GSM302864	Homo sapiens	64	Male	CT
GSE12021	GSM302866	Homo sapiens	88	Female	CT
GSE12021	GSM302870	Homo sapiens	65	Male	CT
GSE12021	GSM303522	Homo sapiens	53	Male	CT
GSE12021	GSM303523	Homo sapiens	29	Female	CT
GSE12021	GSM303525	Homo sapiens	17	Male	CT
GSE12021	GSM303531	Homo sapiens	39	Male	CT
GSE12021	GSM303533	Homo sapiens	36	Male	CT
GSE12021	GSM302880	Homo sapiens	71	Female	OA
GSE12021	GSM302930	Homo sapiens	76	Female	OA
GSE12021	GSM303326	Homo sapiens	61	Female	OA
GSE12021	GSM303341	Homo sapiens	75	Female	OA
GSE12021	GSM303356	Homo sapiens	78	Male	OA
GSE12021	GSM303358	Homo sapiens	64	Male	OA
GSE12021	GSM303360	Homo sapiens	71	Female	OA
GSE12021	GSM303362	Homo sapiens	80	Female	OA
GSE12021	GSM303370	Homo sapiens	66	Female	OA
GSE29746	GSM737470	Homo sapiens	21	Male	CT
GSE29746	GSM737471	Homo sapiens	40	Male	CT
GSE29746	GSM737472	Homo sapiens	58	Female	CT
GSE29746	GSM737473	Homo sapiens	68	Female	CT
GSE29746	GSM737474	Homo sapiens	79	Male	CT
GSE29746	GSM737475	Homo sapiens	46	Female	CT
GSE29746	GSM737476	Homo sapiens	84	Male	CT
GSE29746	GSM737477	Homo sapiens	57	Male	CT
GSE29746	GSM737478	Homo sapiens	58	Female	CT
GSE29746	GSM737479	Homo sapiens	56	Female	CT
GSE29746	GSM737480	Homo sapiens	82	Female	CT
GSE29746	GSM737481	Homo sapiens	80	Male	OA
GSE29746	GSM737482	Homo sapiens	75	Male	OA
GSE29746	GSM737483	Homo sapiens	66	Female	OA
GSE29746	GSM737484	Homo sapiens	80	Male	OA
GSE29746	GSM737485	Homo sapiens	71	Female	OA
GSE29746	GSM737486	Homo sapiens	56	Female	OA
GSE29746	GSM737487	Homo sapiens	73	Female	OA
GSE29746	GSM737488	Homo sapiens	76	Male	OA
GSE29746	GSM737489	Homo sapiens	74	Female	OA
GSE29746	GSM737490	Homo sapiens	80	Male	OA
GSE29746	GSM737491	Homo sapiens	72	Female	OA
GSE55457	GSM1337304	Homo sapiens	61	Male	CT
GSE55457	GSM1337305	Homo sapiens	64	Male	CT
GSE55457	GSM1337306	Homo sapiens	78	Female	CT
GSE55457	GSM1337307	Homo sapiens	65	Male	CT
GSE55457	GSM1337308	Homo sapiens	53	Male	CT
GSE55457	GSM1337309	Homo sapiens	68	Male	CT
GSE55457	GSM1337310	Homo sapiens	29	Female	CT
GSE55457	GSM1337311	Homo sapiens	17	Male	CT
GSE55457	GSM1337312	Homo sapiens	39	Male	CT
GSE55457	GSM1337313	Homo sapiens	36	Male	CT
GSE55457	GSM1337327	Homo sapiens	77	Female	OA
GSE55457	GSM1337328	Homo sapiens	71	Female	OA
GSE55457	GSM1337329	Homo sapiens	76	Female	OA
GSE55457	GSM1337330	Homo sapiens	61	Female	OA
GSE55457	GSM1337331	Homo sapiens	75	Female	OA
GSE55457	GSM1337332	Homo sapiens	78	Male	OA
GSE55457	GSM1337333	Homo sapiens	69	Male	OA
GSE55457	GSM1337334	Homo sapiens	71	Female	OA
GSE55457	GSM1337335	Homo sapiens	80	Female	OA
GSE55457	GSM1337336	Homo sapiens	66	Female	OA
GSE55235	GSM1332201	Homo sapiens	NA	NA	CT
GSE55235	GSM1332202	Homo sapiens	NA	NA	CT
GSE55235	GSM1332203	Homo sapiens	NA	NA	CT
GSE55235	GSM1332204	Homo sapiens	NA	NA	CT
GSE55235	GSM1332205	Homo sapiens	NA	NA	CT
GSE55235	GSM1332206	Homo sapiens	NA	NA	CT
GSE55235	GSM1332207	Homo sapiens	NA	NA	CT
GSE55235	GSM1332208	Homo sapiens	NA	NA	CT
GSE55235	GSM1332209	Homo sapiens	NA	NA	CT
GSE55235	GSM1332210	Homo sapiens	NA	NA	CT
GSE55235	GSM1332211	Homo sapiens	NA	NA	OA
GSE55235	GSM1332212	Homo sapiens	NA	NA	OA
GSE55235	GSM1332213	Homo sapiens	NA	NA	OA
GSE55235	GSM1332214	Homo sapiens	NA	NA	OA
GSE55235	GSM1332215	Homo sapiens	NA	NA	OA
GSE55235	GSM1332216	Homo sapiens	NA	NA	OA
GSE55235	GSM1332217	Homo sapiens	NA	NA	OA
GSE55235	GSM1332218	Homo sapiens	NA	NA	OA
GSE55235	GSM1332219	Homo sapiens	NA	NA	OA
GSE55235	GSM1332220	Homo sapiens	NA	NA	OA

### GO/KEGG enrichment and protein-protein interaction (PPI) network analysis of immune disorder genes

Gene ontology (GO) and Kyoto encyclopedia of genes and genomes (KEGG) pathway analysis was performed using the ‘clusterProfile’ package in R software. All pathways with statistical significance (p < 0.05) were retained. PPI network analysis was performed under the guidance of the String website, which is an open-access website exploring functional protein associations based on the genes we input.

### Identification of hubs genes of SID in OA synovium

Differential expression analysis was performed between OA and control (CT) group, and the differential expressed genes of SID in OA synovium was used for next analysis. The ‘ggplots2’, ‘heatmap’, and ‘ggpubr’ packages were used to create volcano, heatmap clustering, and Box plots, respectively. Subsequently, a random forest classifier consisting of 1000 decision trees was constructed, and ten-fold cross-validation was performed using the ‘randomForest’ R package. The potential hub genes of SID were ranked using the random forest function and the forest plot was drawn using the ‘forestplot’ package. The support vector machine recursive feature elimination (SVM-RFE) was used to remove features with relatively low prediction values in each iteration. Therefore, potential hub genes were ranked from most to least important. The intersection of the top 10 potential hub genes retained by random forest and SVM-RFE analyses was identified as the potential hub genes.

To verify the expression of potential hub genes of SID in OA synovium, we collected the synovial tissues from 39 OA patients who underwent knee joint replacement surgery and 7 normal individuals with acute anterior cruciate ligament (ACL) rupture who underwent ACL reconstruction (testing set). The study was approved by the Ethics Committee of the Third Affiliated Hospital of Anhui Medical University (Approval Number: 2023-022-01), and all patients signed the tissue collection informed consent form. The RT-qPCR was conducted as manufacturer’s instructions. Briefly, total RNA from synovial tissue was extracted using TRIzol reagent (Invitrogen, USA). The RNA concentration was detected with a NanoDrop spectrophotometer. The RNA samples were transcribed into cDNA using the PrimeScript RT reagent kit and then amplified using the TB Green Premix Ex Taq II reagent kit (TaKaRa, Japan). Primer sequences of mRNA were shown in (Table S1). The primers of all mRNAs were purchased from Sangon Biotech https://www.sangon.com/ (China). GAPDH was used as an endogenous control to estimate gene expression levels, and data analysis was performed by standardizing the relative expression levels. The 2^−ΔΔCT^ method was used for data analysis. Each sample was checked three times.

### Construction of OA diagnostic model

Based on the expression of hub genes, we calculated the area under the receiver operator characteristic (ROC) curve to evaluate whether the identified hub genes of SID have diagnostic value in OA. Using ‘ROCR’ package, each point on the ROC curve was generated by training a classifier on all included samples. Then, a standard bootstrap procedure further verified the area under the ROC curve (AUC). Nomogram was conducted to predict the possibility of OA. The calibration curve was drawn to present the stability of the model, and the decision curve analysis was generated to demonstrate whether the constructed model is beneficial to OA patients. All the results were re-verified based on collected clinic data.

### Correlation between hub gene expression and immune microenvironment in OA synovium

The CIBERSORT algorithm (https://cibersortx.stanford.edu/) was used to calculate the infiltrative degree of 22 types of immune-related cells in OA synovium^24^. Using the ‘relative’ and ‘absolute’ methods available on the analysis website CIBERSORT (https://cibersortx.stanford.edu/), the percentage of local immune cell infiltration in OA synovial tissue was analyzed by the CIBERSORT module. Gene expression deconvolution was performed on RNAseq TPM level data of clinical samples. Based on CIBERSORT analysis, we derive the correlation of verified hub genes and 22 immune-related cell infiltration. Then, the ‘MCP-counter’ package quantified the relationship between verified hub genes and the absolute abundance of 9 immune cells in the peripheral blood of OA.

### Consensus clustering and WGCNA analysis

Unsupervised consensus clustering analysis was performed using the “ConsensusClusterPlus” software. The maximum cluster gene number in OA samples was set to 10. The top 5000 most variable genes represented by absolute deviation median were used for sample clustering. Unsupervised consensus clustering was used to cluster OA samples and the optimal number of clusters was selected. In each of the 1000 resampling iterations of the consensus clustering, ward linkage was used as the clustering method and Euclidean distance was used as the distance metric. Weighted gene co-expression network analysis (WGCNA) focuses on gene sets rather than individual gene expression and is a common method for understanding gene correlation patterns between different phenotype traits^25,26^. In the WGCNA analysis, OA expression data can be used to build a powerful scale-free network to identify hub biomarkers for mechanism evaluation and clinical diagnosis. First, the expression of OA synovial genes was standardized. The matrix was then converted into an adjacency matrix using the power adjacency function, which represented the strength of the connection between any two genes. The soft-thresholding parameter for the power adjacency function was selected based on the scale-free topology (SFT) criterion, which is a necessary condition for network construction. The best threshold parameter value with a model fitting saturation > 0.85 was accepted based on the SFT criterion recommendation. The “tree” method of “deepSplit” was used to determine the modules, which identified modules with at least 10 genes. Each module was characterized by feature genes, which were generated by WGCNA. Fisher’s exact test was used to detect enriched genes in each module of OA synovial tissue. Gene set enrichment analysis (GSEA) was used to evaluate the enrichment score of DEGs among the three clusters.

### Statistical analysis

Statistical analysis was performed using SPSS software (version 23.0). All raw data processing and analysis were performed using R software (version 4.2.1). Kruskal-­‐Walli’s test or Wilcoxon test was used to detect significant differences between the CT group and the OA group. P < 0.05 was considered statistically significant.

## Results

### Identification and enrichment analysis of 32 immune disorder genes in OA

**Fig. 2:**
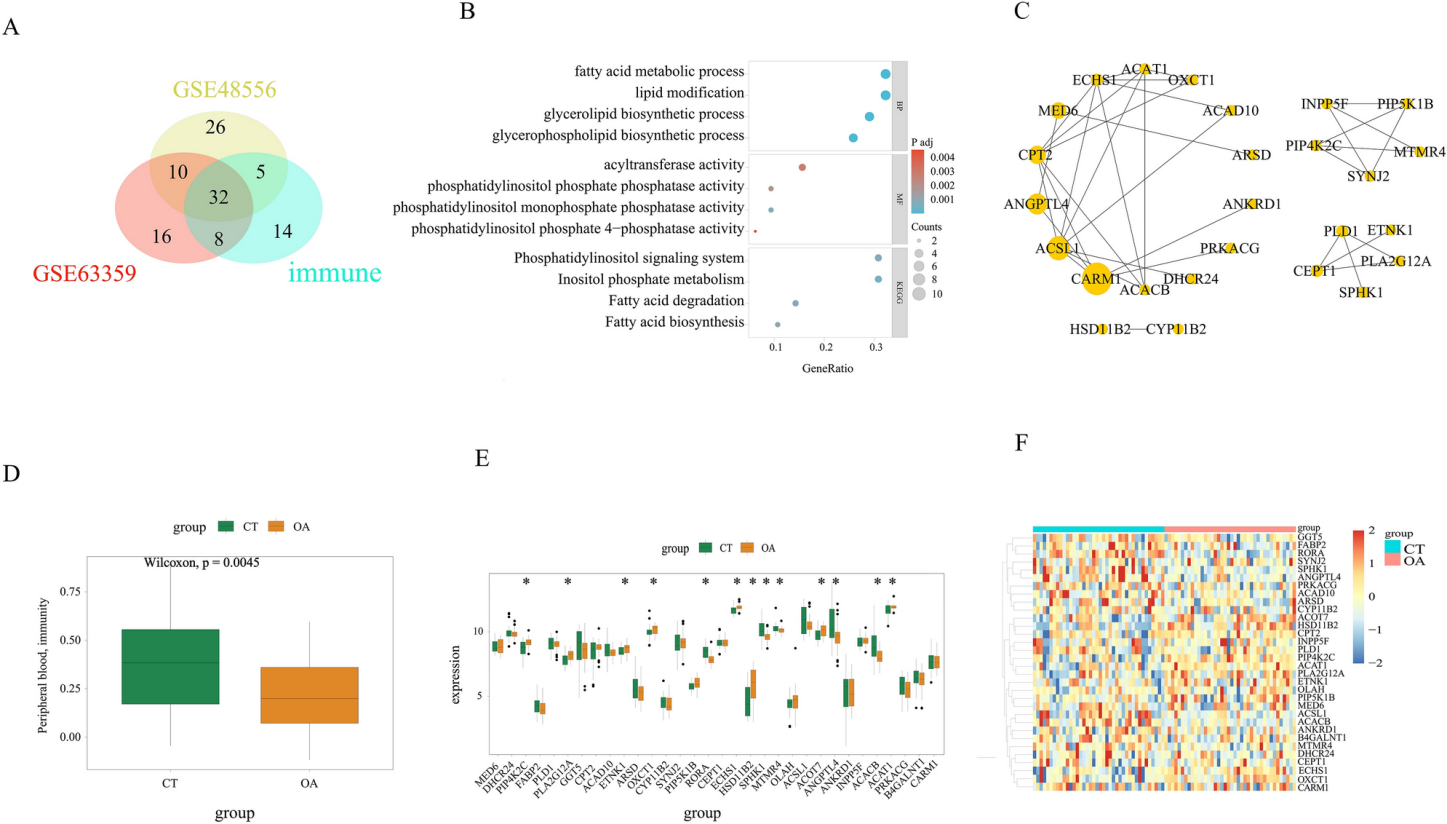
Identification of synovial SID-related genes in OA. (**A**) Identification of SID-related genes both in two peripheral blood components and immune. (**B**) GO/KEGG analysis of identified SID-related genes. (**C**) Protein-protein interaction (PPI) network analysis of identified SID-related genes. The larger the yellow oval, the stronger the interaction of the node, with a corresponding increase in the number of edges connected to it. Six genes without interactions were removed. (**D**) Expression of SID-related genes between OA and healthy control. (**E**) Identification of synovial SID-related genes in OA. (**F**) Heat map of expression heterogeneity of synovial SID-related genes between OA and healthy control.

We performed differential gene expression analysis based on blood-related transcriptome profiles (GSE48556, GSE63359) in patients with osteoarthritis (OA). Utilizing Venn analysis, we successfully identified 32 immune disorder-related genes that exhibited differential expression in the peripheral blood of OA patients (Fig. 2A, Table S2). Subsequently, a comprehensive GO/KEGG analysis was conducted on these 32 immune disorder-related genes (Fig. 2B, Table S3), revealing their significant enrichment in bioprocesses associated with lipid metabolism, such as “fatty acid metabolic process” and “lipid modification”. Additionally, the molecular function of the immune disorder-related genes showed a significant enrichment in “acyltransferase activity”. Among the KEGG pathways, the most prominently enriched pathways were “Phosphatidylinositol signaling system” and “Inositol phosphate metabolism”. The protein–protein interaction (PPI) network of the 32 immune disorder-related genes is depicted in (Fig. 2C).

After addressing batch effects in four transcriptome profiles related to OA synovium (Fig. S1), we applied these 32 immune disorder-related genes for differential expression analysis in OA synovium. Our findings revealed a significant overall down-regulation in the expression levels of immune disorder-related genes (Fig. 2D). Furthermore, we identified 13 differentially expressed genes (DEGs) associated with immune disorders in OA synovium (Fig. 2E). Heatmap analysis based on an integrated dataset demonstrated heterogeneity in the expression patterns of these 32 genes between healthy (CT) and OA synovium samples (Fig. 2F).

### Three SID-related hub genes can predict OA occurrence

**Fig. 3:**
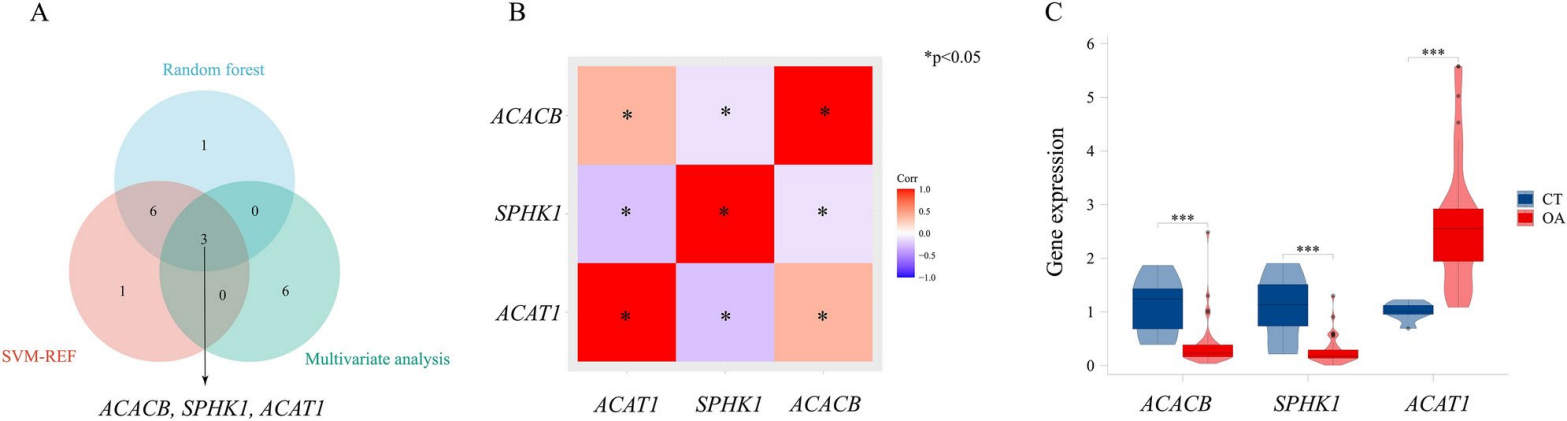
Identification of hub genes of SID in OA. (**A**) Three hub genes were identified by random forest, SVM-RFE, and multivariate analysis. (**B**) Correlation between three expressed hub genes in OA synovium. (**C**) Expression levels of the three hub genes were quantified by qPCR analysis in health and OA synovium (n = 7, n = 39).

We employed random forest analysis (Fig. S2A) and SVM-RFE algorithm (Fig. S2B) to rank the 32 immune disorder-related genes based on their importance in OA synovium. The overlapping genes obtained from the top 10 ranked genes by both algorithms were further subjected to multivariable logistic regression analysis (Fig. S2C). Through Venn analysis, we ultimately identified three hub genes of synovial immune disorder (SID): Acetyl-CoA Acetyltransferase 1 (*ACAT1*), Sphingosine Kinase (*SPHK1*), and Acetyl-CoA Carboxylase Beta (*ACACB*) (Fig. 3A). Notably, these three genes exhibited a significant correlation in their expression levels (Fig. 3B).

**Fig. 4:**
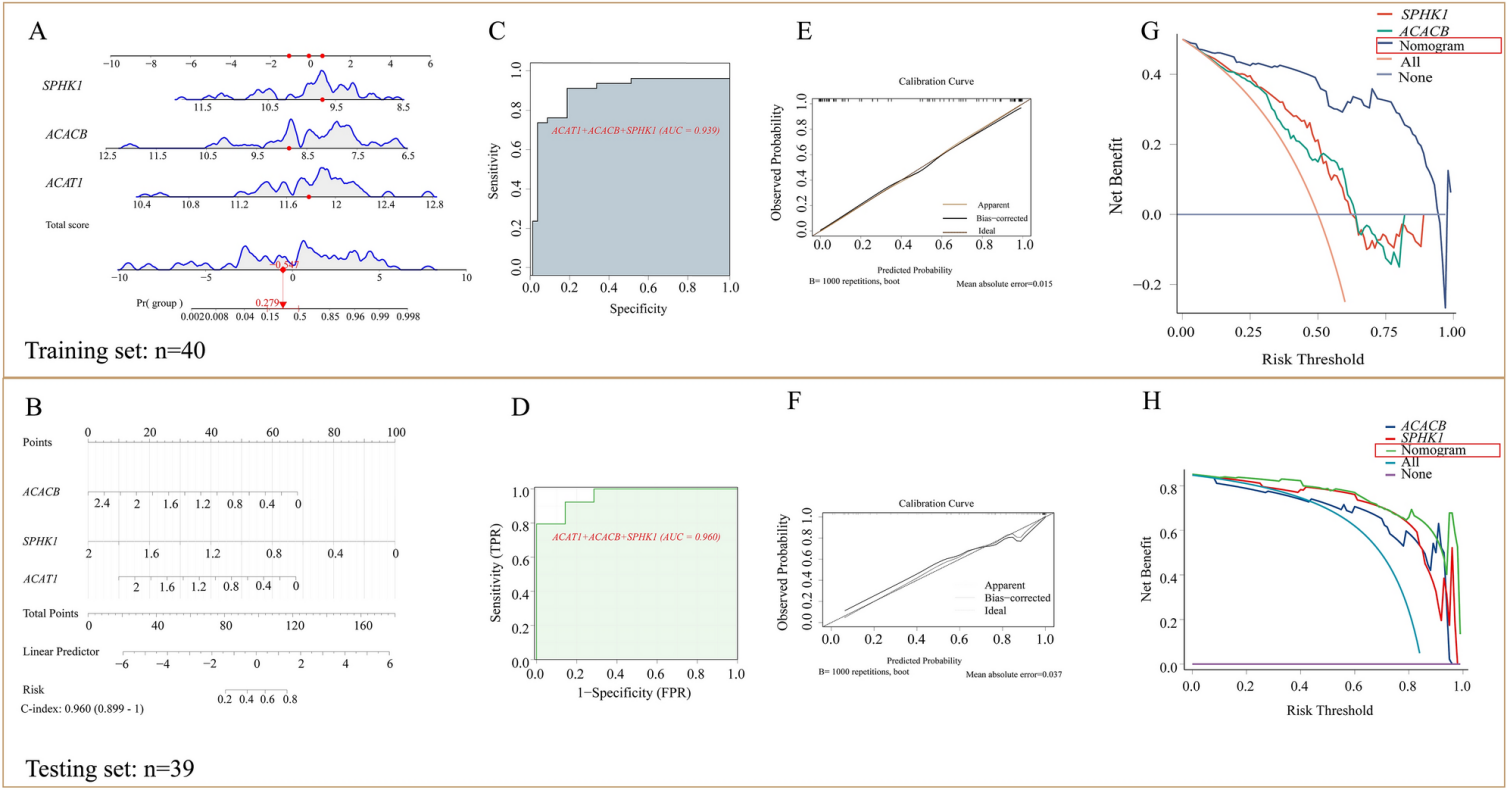
Construction and validation of nomogram model. (**A**) Utilized three hub genes of SID, we generated nomogram for the training set and (**B**) testing set. (**C**) Receiver operating characteristic (ROC) curve of nomogram for OA diagnosis accuracy in training set and (**D**) testing set. (**E**) Calibration curve of the diagnostic model in training set and (**F**) testing set. (**G**) Decision curve analysis (DCA) in training set and (**H**) testing set reveals that nomogram model will be more benefits for patients with OA.

Subsequently, we quantified the expression levels of these three hub genes in OA synovium using RT-qPCR (Fig. 3C) in testing set. Among them, two genes were found to be down-regulated while one gene was up-regulated. Based on the expression levels of these three hub genes in the training and testing sets, we constructed a diagnostic model. The nomogram generated for the training set (Fig. 4A) and testing set (Fig. 4B) demonstrated that the probability of OA occurrence could be determined by the total score calculated using the expression levels of the identified hub genes. The AUC values indicated that the diagnostic model had excellent predictive performance for OA occurrence in both training set (Fig. 4C, AUC = 0.939) and testing set (Fig. 4D, AUC = 0.960). Additionally, the well-calibrated calibration curves further confirmed the reliability of the predictive model (Fig. 4E,F). Compared to individual hub genes, the diagnostic model incorporating these three SID-related hub genes provided improved benefits and diagnostic ability for OA patients, as evidenced by the higher AUC values (Fig. 4G,H, Fig. S2D). We validated the reliability of the ROC curve using bootstrap sampling from OA samples (n = 100 bootstraps) (Fig. S2E), and the distribution ranges of AUC, sensitivity, and specificity for the diagnostic model are presented in (Fig. S2F–H), respectively.

### The correlation between SID-related hub genes and immune pattern in OA synovium

**Fig. 5:**
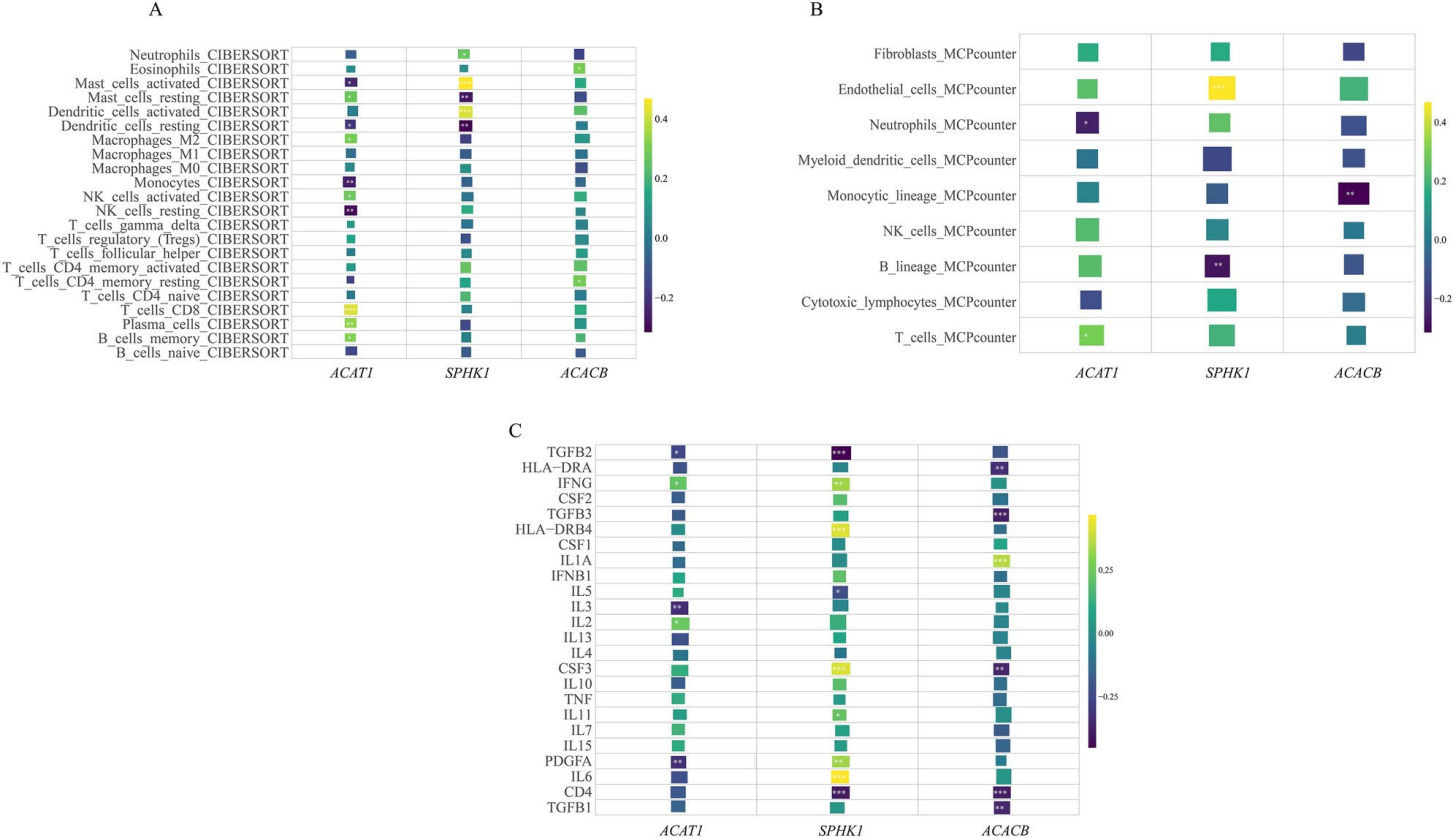
The correlation between three hub genes expression and immune model. (**A**) Correlation between hub genes expression and 22 immune-related cell infiltration (CIBERSORT). (**B**) Correlation between hub genes expression and the abundance of 9 immune-related cells (MCP-counter). (**C**) Correlation between hub genes expression and the level of 24 inflammatory cytokines. *p < 0.05, **p < 0.01, ***p < 0.001.

We explored the correlation between the expression levels of three hub genes immune cell infiltration, and inflammatory cytokines using CIBERSORT and mcp-counter analysis (Fig. 5). We found that *ACAT1* was significantly correlated with the levels of 10 out of 22 immune cell infiltrations, 5 out of 24 inflammatory cytokines, and 2 out of 9 immune cell populations. For *SPHK1*, we observed significant correlations between its expression level and 5 out of 22 immune cell infiltrations, 9 out of 24 inflammatory cytokines, and 2 out of 9 immune cell populations. The expression level of *ACACB* was significantly correlated with 2 out of 22 immune cell infiltrations, 6 out of 24 inflammatory cytokines, and negatively correlated with the abundance of monocytes.

### Comparison of age, gender, and immune characteristics among the three groups

**Fig. 6:**
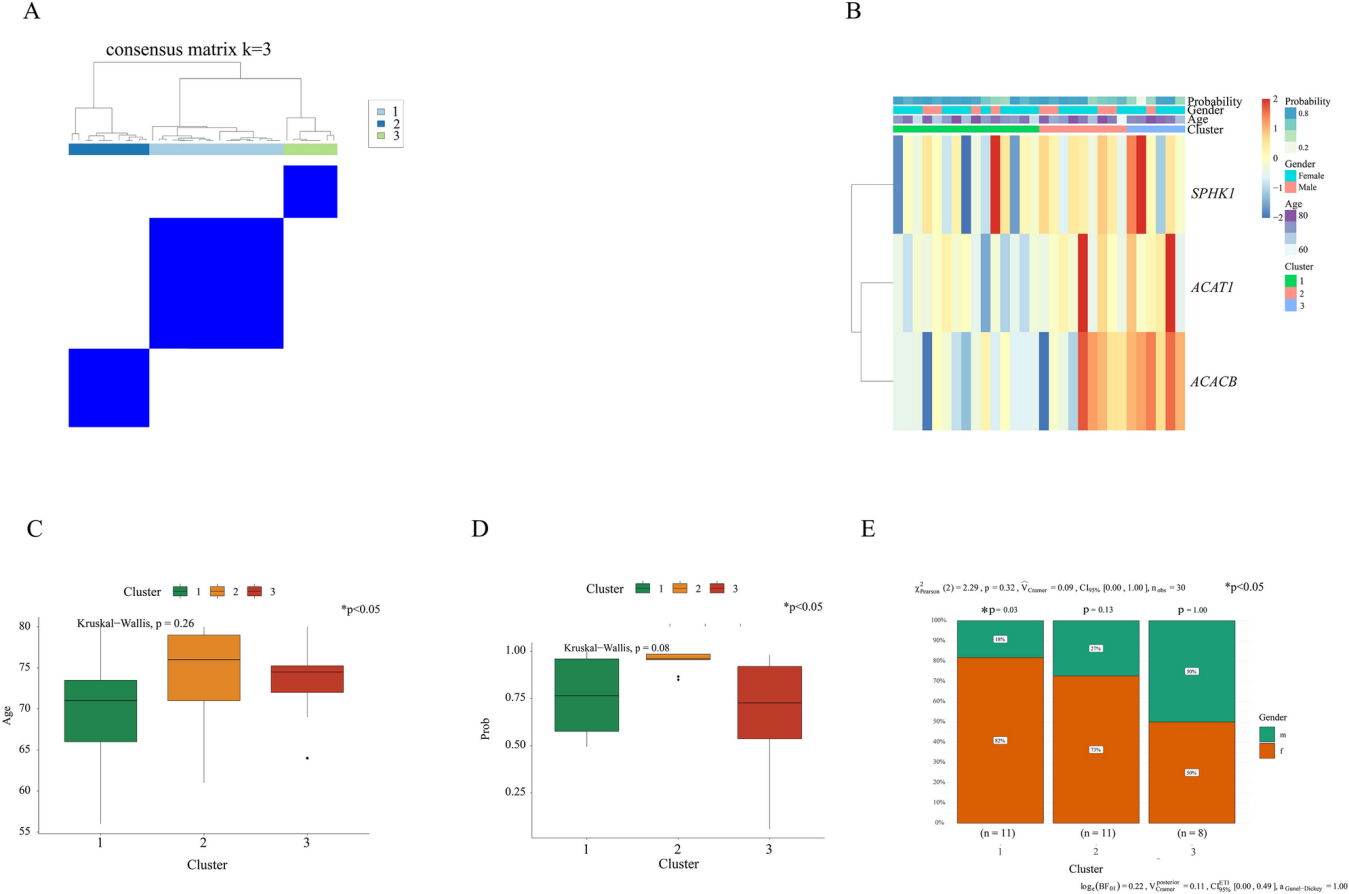
The results of unsupervised consensus clustering based on identified hub genes. (**A**) Consensus matrix plots depicting consensus values on a white-to-blue color scale ordered by consensus clustering when three clusters were selected. (**B**) The heterogeneity of gene expression is related to age and gender. Age (**C**) and age probability (**D**) difference between three clusters. (**E**) Gender difference between three clusters.

**Fig. 7:**
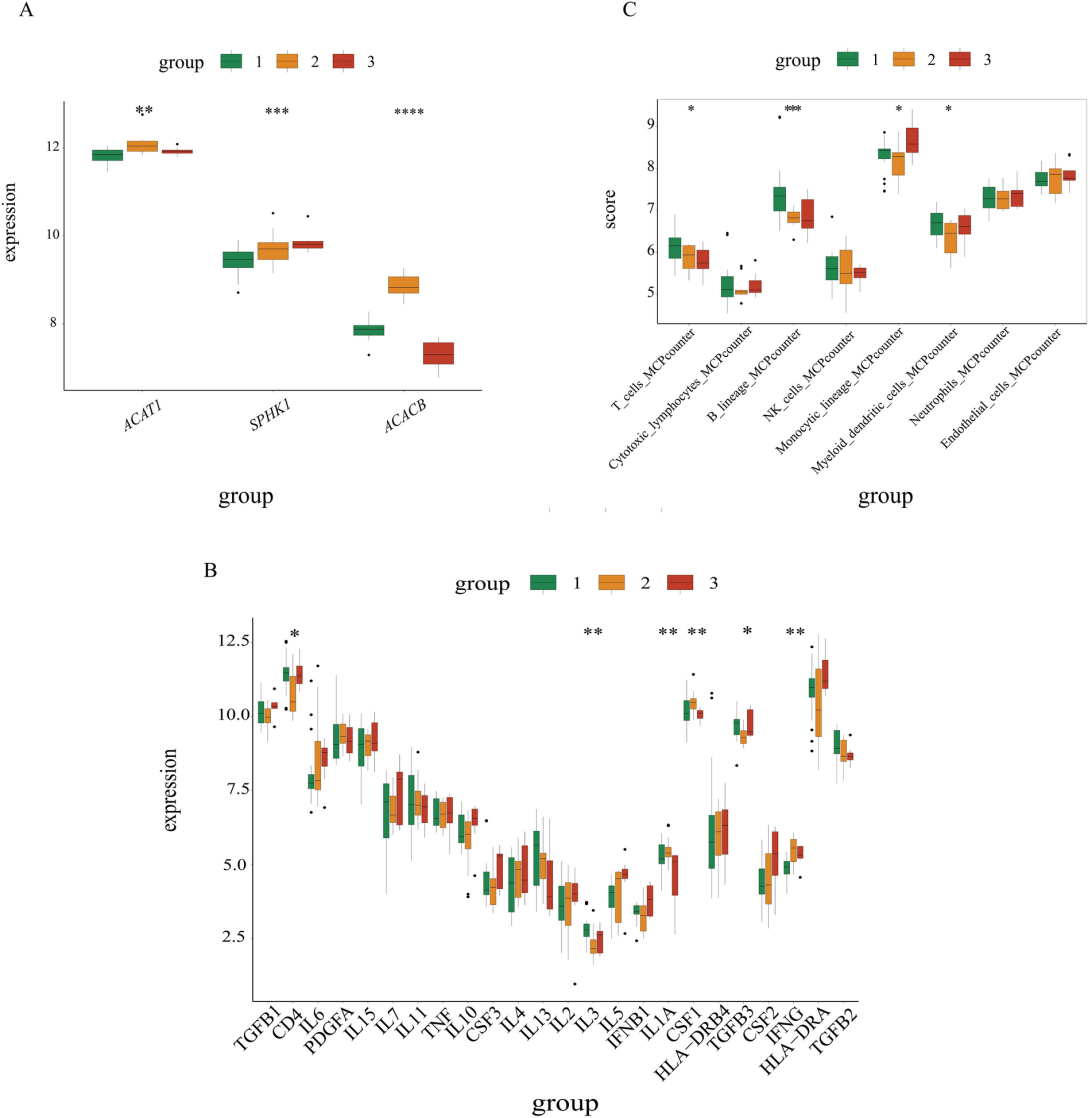
(**A**) Expression differences of three hub genes between three clusters. (**B**)The difference of immune cells abundance between three clusters. (**C**) The difference of inflammatory factor levels between three clusters.

Utilizing consensus cluster analysis based on the expression levels of the three hub genes, we successfully clustered the OA patients into three distinct groups (Fig. 6A). To examine the relationship between the hub genes and clinical factors, we performed analyses using the “ggpubr” package in R software. As shown in (Fig. 6B), there were significant heterogeneities observed in the expression of the three hub genes, as well as gender, age, and the likelihood of OA occurrence among the three clusters. Although no significant difference in age was observed across all three clusters (Fig. 6C), there was a variation in the likelihood of OA occurrence (Fig. 6D, p < 0.05). Notably, the number of female patients appeared to be higher than that of males in the younger group (Fig. 6E, cluster 1, p = 0.03). In the elderly group (cluster 2), the expression levels of *ACAT1, SPHK1, ACACB*, CSF1, and IFNG were upregulated, while CD4, IL3, IL1A, and TGFB3 exhibited downregulation (Fig. 7A,B). Additionally, T cells, B cells, monocytes, and myeloid dendritic cells were found to be decreased in cluster 2 (Fig. 7C).

### Construction of co-expression network

**Fig. 8:**
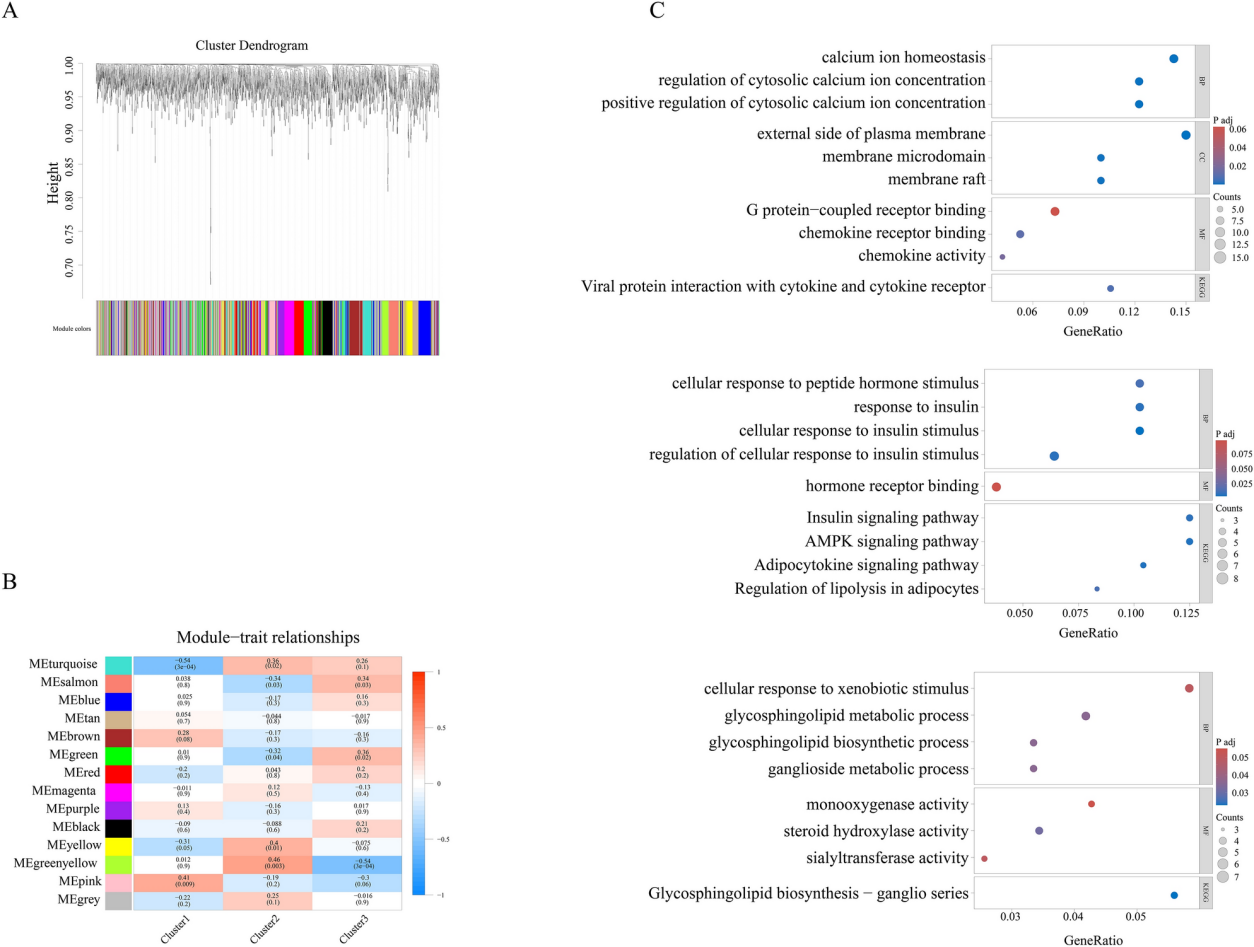
Identification of key modules correlated with three clusters through WGCNA. (**A**) Clustering dendrogram of genes based on topological overlapping. (**B**) Heatmap of the correlation between module eigengenes and three clusters. (**C**) GO/KEGG analysis for three clusters. *p < 0.05, **p < 0.01, ***p < 0.001.

A total of 40 osteoarthritis (OA) samples underwent quality control analysis, and all samples were included in subsequent analyses (Fig. S3A). Co-expression modules were constructed using the dynamic tree cutting methodology, and an unscaled network was generated with a soft threshold value of 5 (Fig. S3B). The identification of statistically significant co-expression modules was performed using weighted gene co-expression network analysis (WGCNA) based on optimal dynamic tree cutting and hierarchical clustering (Fig. 8A,B). Notably, the pink module showed the strongest positive correlation with cluster 1, while the greenyellow module exhibited a positive correlation with cluster 2, and the green module was positively correlated with cluster 3. Subsequently, GO/KEGG analysis was conducted for genes within these three modules (Fig. 8C). The genes in the pink module were mainly involved in the biological process of “positive regulation of cytosolic calcium ion concentration,” while the genes in the greenyellow module were significantly enriched in the KEGG pathway of “AMPK signaling pathway.” Within the green module, the genes primarily participated in the biological process of “glycosphingolipid metabolic process” (Fig. 8C). Furthermore, we explored the enriched pathways using gene set enrichment analysis (GSEA) based on differentially expressed genes (DEGs) among the three clusters (Fig. S4). Our findings revealed that the “phospholipase c activating G protein coupled receptor signaling pathway,” “adaptive immune response,” and “leukocyte mediated cytotoxicity” showed a positive correlation with cluster 1 and a negative correlation with cluster 2.

## Discussion

OA is increasingly recognized as a low-grade inflammatory condition, primarily characterized by immune dysregulation and inflammatory responses in the synovium. While previous studies have identified immune-related genes associated with osteoarthritis^27^, the identification of SID-related genes in osteoarthritic synovial tissue and their corresponding roles remain unexplored. In this study, we characterized 32 SID genes, conducted functional enrichment and immune infiltration analyses, and identified three potential biomarkers that can distinguish osteoarthritis.

Bioinformatics analysis is a widely utilized approach to explore key genes and pathways associated with diseases. For instance, Mahima et al. employed various bioinformatics methods to identify key biomolecules involved in bone metastasis in breast^28^ and prostate^29^ cancer. Van et al. demonstrated a difference in lipid metabolites between OA synovium and healthy control group^30^. In our study, we identify three SID-related hub genes that can predict OA occurrence. And 32 SID genes were primarily enriched in biological processes such as “fatty acid metabolic process” and “lipid modification”. Furthermore, the most significantly enriched KEGG pathway was the “Phosphatidylinositol signaling system”. This suggests a potential interaction between lipid metabolism and immune disorders in the peripheral blood of OA patients^11^. Acyltransferase activity, which regulates disease progression, has been proposed as a potential biomarker for OA^31^. Of interest, the “acyltransferase activity” was the most significantly enriched molecular function among the SID-related genes. Further studies are needed to explore the modulation of acyltransferase activity in OA^31^.

Studies have reported potential novel biomarkers for diagnosing OA^11,32^. However, the constructed diagnostic models have been limited to identifying DEGs from a single articular component^32^, and reliable SID-related markers are rarely reported. In our study, we utilized bioinformatics and RT-qPCR to identify three hub genes of SID that were differentially expressed in both OA synovium and peripheral blood. Our constructed model suggests the possibility of using SID-related genes to screen for early-onset OA. *SPHK1* enhances inflammatory response and increases the level of inflammatory mediators such as TGFB2 and IL6^33,34^. Reduced *SPHK1* expression is associated with attenuated joint pathology and synovitis progression in invasive arthritis^35^. Conversely, upregulation of *SPHK1* can offer protection against ischemic heart disease and prevent cardiomyocyte death resulting from excessive production of reactive oxygen species^36^. In this study, the *SPHK1* expression was down-regulated and positively correlated with expression level of IL-6 and the abundance of endothelial cells. These results suggest that down-regulated *SPHK1* may reduce inflammation and synovial endothelial cells in OA synovium. The specific impact of *SPHK1* expression in OA synovial immune needs further exploration. Previous studies have shown that IL1A is a main Pro-inflammatory factor which not only can aggravate the development of OA, but also arouse articular pain^37^. Meanwhile, the expression of CSF3 can reduce pain sensation induced by inflammatory factors^38,39^. In the current study, *ACACB* was found to be down-regulated in OA synovium and was positively correlated with IL1A, while being negatively correlated with CSF3. These results were consistent with previous studies. In the present investigation, we observed a down-regulation of *ACAT1* in OA synovium, which was found to be associated with inflammatory factors. However, our current study reveals an up-regulation of *ACAT1* expression in the synovium of patients with osteoarthritis, and a negative correlation with the levels of TGFB2 and IL-3. These factors have been implicated in ameliorating the degeneration of osteoarthritis cartilage and subchondral bone^40,41^. Conversely, *ACAT1* expression exhibited a positive association with IL-2, a cytokine known to promote the progression of OA inflammation^42^. Collectively, these findings suggest that *ACAT1* may serve as a potential risk factor in osteoarthritis.

In this study, we distinguished the 40 OA patients into three clusters (cluster 1, cluster 2 and cluster 3). The cluster 2 (Older than the other two groups) exhibited downregulated relative scores of T cells, B cells, monocytes, and myeloid dendritic cells, indicating potential immune cell suppression in the elderly OA population. Additionally, we identified upregulation of CSF1 and IFNG, along with downregulation of CD4, IL3, IL1A, and TGFB3 in cluster 2. Previous studies have linked low levels of CSF1 to significant alleviation of OA progression^43^, while high levels of IL3 and TGFB3 have been associated with improved synovitis development in OA^12,41^. These results suggest that targeting downregulation of CSF1 or upregulation of IL3 and TGFB3 could be potential therapeutic strategies for elderly patients with OA.

WGCNA analysis revealed that characteristic genes in the pink, greenyellow, and green modules were positively correlated with Cluster 1, Cluster 2, and Cluster 3, respectively. Specifically, the characteristic genes of the pink module primarily participate in the “positive regulation of cytosolic calcium ion concentration,” which is crucial for regulating immune and inflammatory responses^44,45^. Dysregulation of calcium homeostasis has been implicated in synovial inflammation, and calcium ions have shown potential protective and therapeutic effects in osteoarthritis^46,47^. Therefore, future treatment plans for Cluster 1 (younger OA patients) should focus on targeting the regulation of intracellular calcium ion concentration. AMPK, a key regulator of immune cell metabolism and function, plays a critical role in inhibiting inflammatory responses associated with OA^48^. Activating the AMPK signal has also been found to limit the development and progression of the degenerative diseases^49^. In our current study, the characteristic genes of the greenyellow module were enriched in the “AMPK signaling pathway.” Hence, in future treatment plans for Cluster 2 (elderly OA patients), particular attention should be given to targeting the AMPK-related signaling pathway. Lastly, the characteristic genes of the green module were primarily involved in the “glycosphingolipid metabolic process,” which has been shown to inhibit immune responses^50,51^. Degreed the level of glycosphingolipid metabolism has been proposed as a promising approach for treating OA^52^. Therefore, effective treatment strategies for OA patients in Cluster 3 should prioritize targeting the “glycosphingolipid metabolic process.”

Several limitations exist in our study. Several limitations exist in our study that should be acknowledged. Firstly, we did not explore the influence of other variables such as disease duration and anti-OA medications (e.g., corticosteroids and NSAIDs) on inflammatory gene expression. Secondly, although we have identified three SID-related hub genes, whose expression levels can predict the onset of OA and are linked to synovial immunity, we have not investigated their translational levels or the underlying mechanisms associated with OA development. This represents a critical area for future investigation.

## Conclusion

In conclusion, our comprehensive analysis of SID-related genes in OA synovium has revealed three hub genes (*ACAT1*, *ACACB* and *SPHK1*), that hold potential ability for predicting OA occurrence. These hub genes exhibit significant involvement in immune dysregulation within the OA synovium. Furthermore, our findings highlight a strong correlation between the AMPK signaling pathway and elderly OA patients, with a notable decrease in the expression levels of *ACACB* and *SPHK1* observed among older OA patients compared to their younger counterparts. This study provides valuable insights into the exploration of novel biomarkers for OA, contributing to the advancement of future research in this field.

## Supplementary Information

Supplementary Information.

## Data Availability

The datasets used and/or analyzed during the current study are available from the corresponding authors upon reasonable request.
