# Supplementary material for: Integrated bioinformatics and clinical data identify three novel biomarkers for osteoarthritis diagnosis and synovial immune
Source: Sci Rep. 2025 Mar 31;15:10987. doi: 10.1038/s41598-025-95837-7 (PMC11958655; doi:10.1038/s41598-025-95837-7)
Supplement: Supplementary file 1 — Supplementary Information. [file 41598_2025_95837_MOESM1_ESM.pdf]

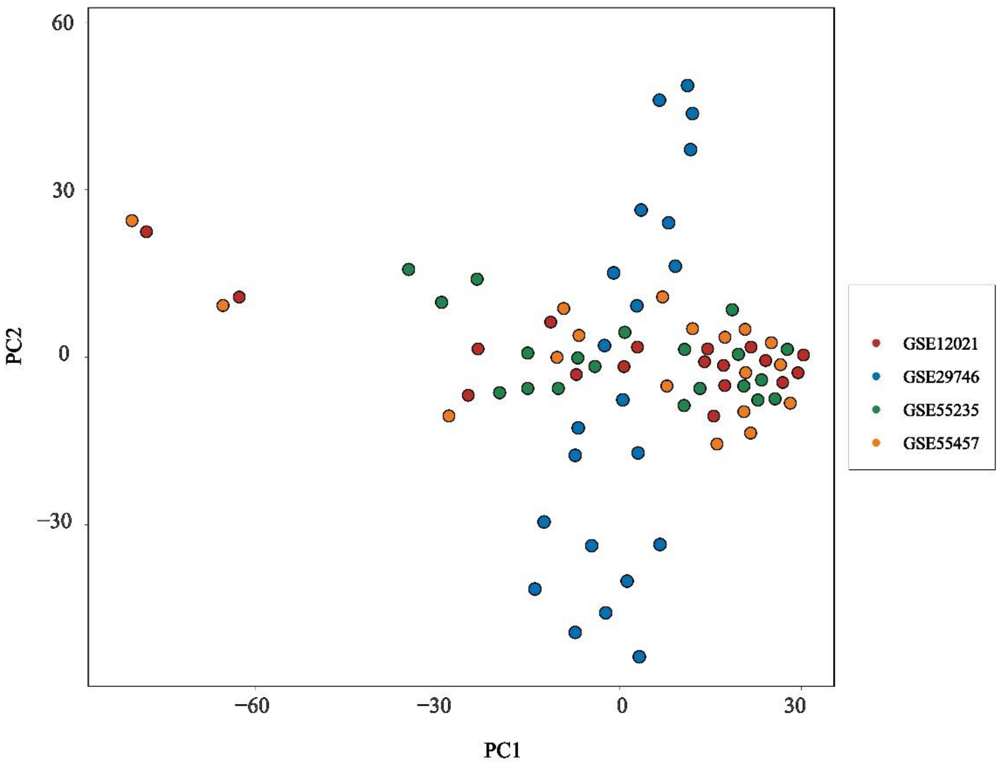

Figure S1: PCA plot of four obtained datasets after batch effects are removed.

A

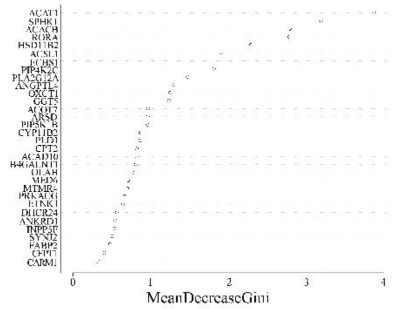

B

SVM-RFE	Rank
ACAT1	1
SPHK1	2
ACACB	3
RORA	4
PIP4K2C	5
PLA2G12A	6
HSD11B2	7
ACOT7	8
ECHS1	9
ANGPTL4	10

D

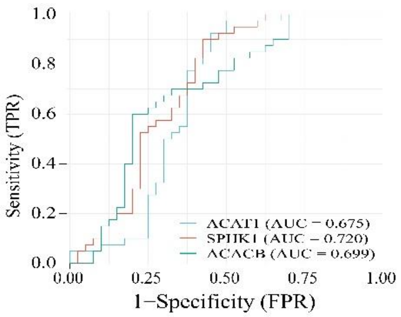

C

*p<0.05

Characteristics	Total(N)	OR(95% CI) Univariate analysis	P value Univariate analysis	OR(95% CI) Multivariate analysis	P value Multivariate analysis
ACAT1 *	80	9.327 (7.764 - 10.891)	0.005	1330.481 (1325.080 - 1335.883)	0.009
ANGPTL4	80	0.543 (0.139 - 0.946)	0.003	0.752 (-0.672 - 2.176)	0.695
ACACB *	80	0.423 (-0.139 - 0.986)	0.003	0.050 (-1.757 - 1.858)	0.001
PLA2G12A	80	4.398 (3.410 - 5.386)	0.003	0.606 (-1.830 - 3.042)	0.687
SPHK1 *	80	0.201 (-0.730 - 1.132)	< 0.001	0.066 (-2.582 - 2.714)	0.044
PIP4K2C	80	7.435 (6.275 - 8.595)	< 0.001	10.353 (7.517 - 13.188)	0.106
ECHS1	80	15.428 (13.649 - 17.206)	0.003	28.218 (24.339 - 32.096)	0.091
HSD11B2	80	1.898 (1.537 - 2.258)	< 0.001	1.241 (0.347 - 2.136)	0.636
RORA	80	0.097 (-1.016 - 1.210)	< 0.001	0.223 (-1.658 - 2.104)	0.118

E

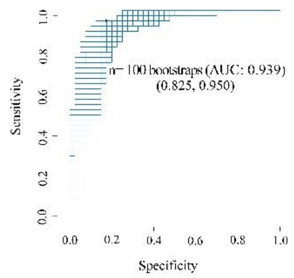

F

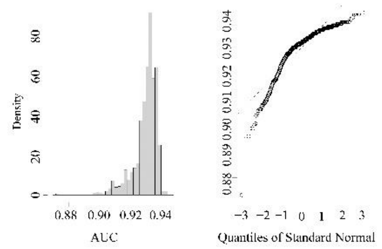

G

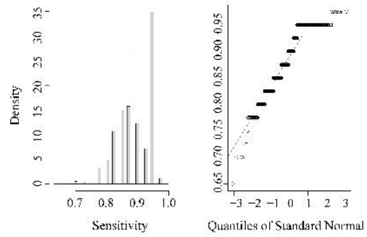

H

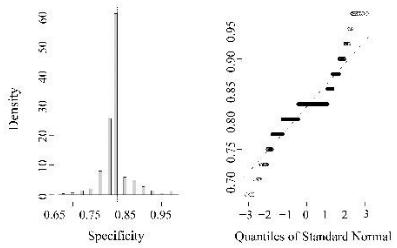

Figure S2: (A) Ranking of gene importance by random forest analysis. (B) Ranking of gene importance by VM recursive feature elimination algorithm. (C) Multivariate logistic regression analysis of top-ranked genes. (D) Construction of ROC curves for the individual diagnosis of the three genes. validation of reliability of ROC model busing the bootstrap procedure (n=1000) (E). Distribution range of AUC(F), sensitivity(G), and specificity (H).

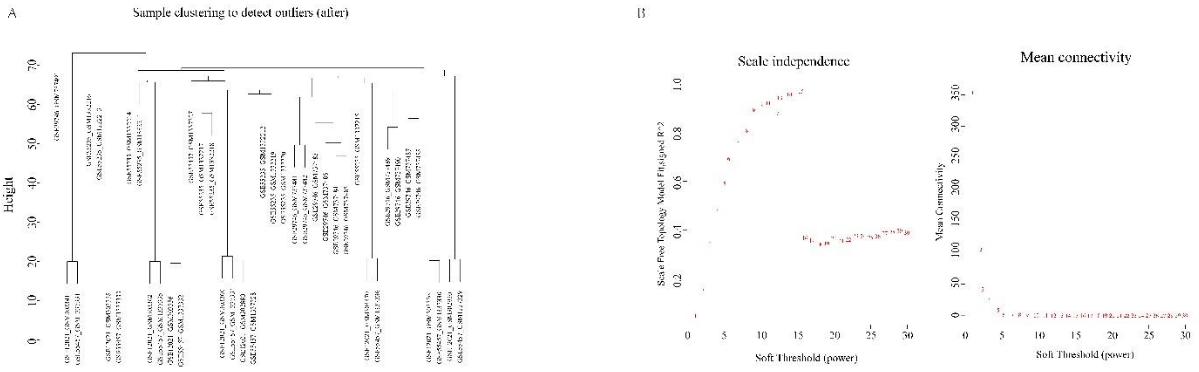

Figure S3: (A) Clustering dendrograms of samples. (B) Analysis of network topologies for various soft-thresholding powers through the scale-free fit index (left) and mean connectivity (right).

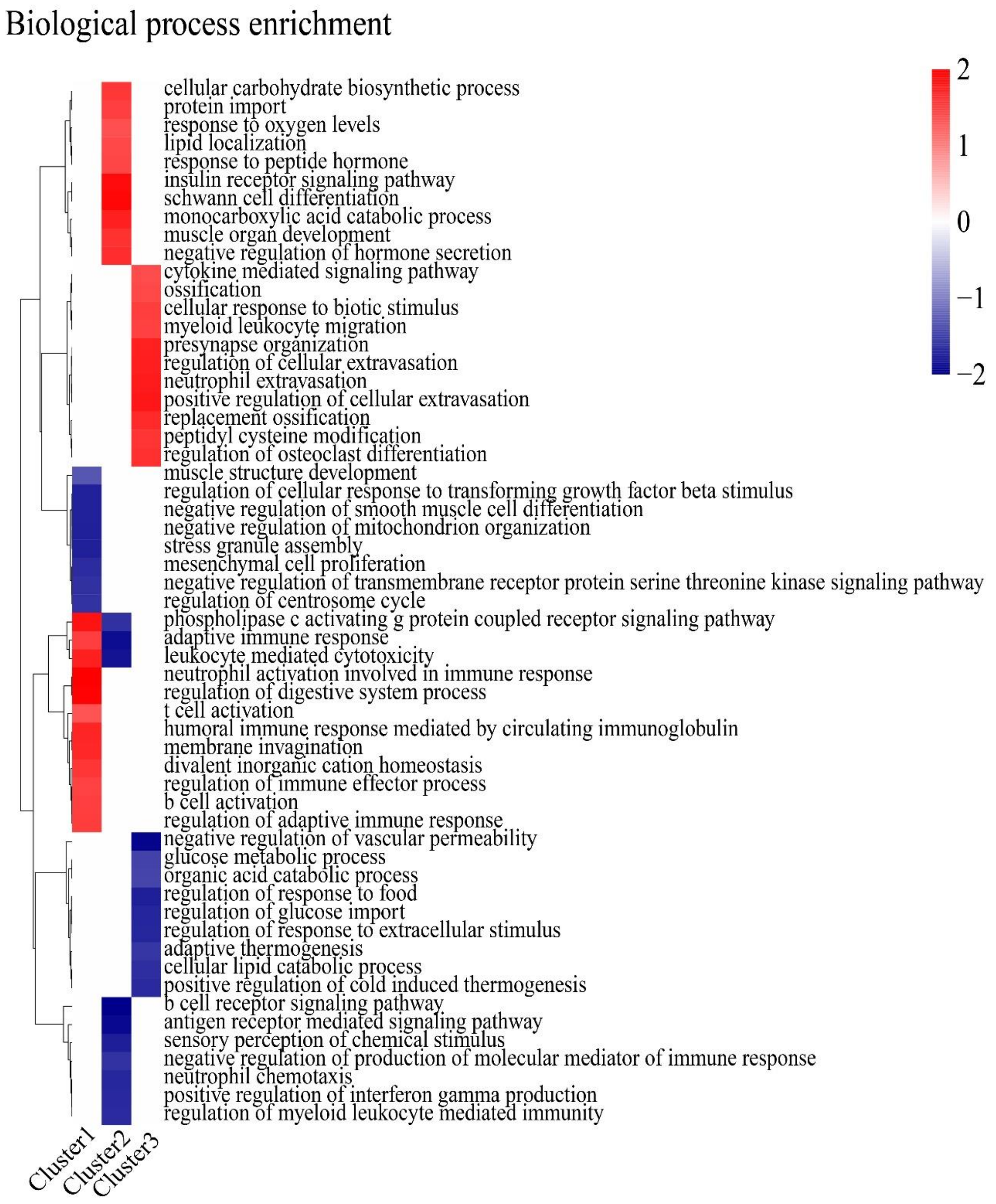

Figure S4: GSEA enrichment analysis of DEGs in three clusters.

Table S1: Primer sequences used in the qRT-PCR experiment in this article

Gene	Forward Sequence (5′-3′)	Reverse Sequence (5′-3′)
ACAT1	TGGAGGAGCTGTTTCTCTGG	GCAAATACTGGCAAGACCGT
ACACB	ATGTTCAGGCAGGCTCTCTT	ATTTCCACCAGGAAGTCGGT
SPHK1	CCTGGGCAGTGAGATGTTTG	ACCACGGGCACATATACCAA
GAPDH	CCTGGCACCCAGCACAAT	GGGCCGGACTCGTCATAC

Table S2: Full names and its abbreviation of the 32 immune disorder-related genes

Genes	Full names	Genes	Full names
FABP2	Fatty acid binding protein 2	SYNJ2	Synaptojanin 2
ACOT7	Acyl-CoA thioesterase 7	PLA2G12A	Phospholipase A2 group XIIA
PIP4K2C	Phosphatidylinositol-5-phosphate 4-kinase type 2 gamma	CARM1	Coactivator associated arginine methyltransferase 1
PRKACG	Protein kinase cAMP-activated catalytic subunit gamma	ECHS1	Enoyl-CoA hydratase, short chain 1
OXCT1	3-oxoacid CoA-transferase 1	MED6	Mediator complex subunit 6
CYP11B2	Cytochrome P450 family 11 subfamily B member 2	MTMR4	Myotubularin related protein 4
ACAT1	Acetyl-CoA acetyltransferase 1	ANGPTL4	Angiopoietin like 4
SPHK1	Sphingosine kinase 1	CPT2	Carnitine palmitoyltransferase 2
HSD11B2	Hydroxysteroid 11-beta dehydrogenase 2	INPP5F	Inositol polyphosphate-5-phosphatase F
ACSL1	Acyl-CoA synthetase long chain family member 1	PLD1	Phospholipase D1
PIP5K1B	Phosphatidylinositol-4-phosphate 5-kinase type 1 beta	ETNK1	Ethanolamine kinase 1
ANKRD1	Ankyrin repeat domain 1	CEPT1	Choline/ethanolamine phosphotransferase 1
ACAD10	Acyl-CoA dehydrogenase family member 10	ACACB	Acetyl-CoA carboxylase beta
GGT5	Gamma-glutamyltransferase 5	RORA	RAR related orphan receptor A
ARSD	Arylsulfatase D	OLAH	Oleoyl-ACP hydrolase
B4GALNT1	Beta-1,4-N-acetyl-galactosaminyltransferase 1	DHCR24	24-dehydrocholesterol reductase

Table S3: 32 peripheral blood immune-related genes GO-KEGG analysis.

Ontology	ID	Description	GeneRatio	BgRatio	pvalue	p.adjust
BP	GO:0030258	lipid modification	10/31	216/18800	1.17e-12	9.47e-10
BP	GO:0045017	glycerolipid biosynthetic process	9/31	253/18800	1.96e-10	7.92e-08
BP	GO:0006631	fatty acid metabolic process	10/31	395/18800	4.48e-10	1.21e-07
BP	GO:0046474	glycerophospholipid biosynthetic process	8/31	214/18800	1.56e-09	3.05e-07
BP	GO:0016042	lipid catabolic process	9/31	327/18800	1.88e-09	3.05e-07
MF	GO:0052744	phosphatidylinositol monophosphate phosphatase activity	3/32	19/18410	4.54e-06	0.0006
MF	GO:0052866	phosphatidylinositol phosphate phosphatase activity	3/32	34/18410	2.75e-05	0.0020
MF	GO:0016746	acyltransferase activity	5/32	244/18410	5.9e-05	0.0028
MF	GO:0034596	phosphatidylinositol phosphate 4-phosphatase activity	2/32	10/18410	0.0001	0.0045
MF	GO:0052629	phosphatidylinositol-3,5-bisphosphate 3-phosphatase activity	2/32	11/18410	0.0002	0.0045
KEGG	hsa00562	Inositol phosphate	5/28	73/8164	4.17e-06	0.0005

Ontology	ID	Description	GeneRatio	BgRatio	pvalue	p.adjust
		metabolism				
KEGG	hsa00071	Fatty acid degradation	4/28	43/8164	1.25e-05	0.0007
KEGG	hsa04070	Phosphatidylinositol signaling system	5/28	97/8164	1.69e-05	0.0007
KEGG	hsa00061	Fatty acid biosynthesis	3/28	18/8164	2.85e-05	0.0009
KEGG	hsa01212	Fatty acid metabolism	4/28	57/8164	3.86e-05	0.0010
